# Violence witnessing, perpetrating and victimization in medellin, Colombia: a random population survey

**DOI:** 10.1186/1471-2458-11-628

**Published:** 2011-08-05

**Authors:** Luis F Duque, Nilton E Montoya, Alexandra Restrepo

**Affiliations:** 1 Director PREVIVA, School of Public Health, University of Antioquia. Calle 62 # 52-57, Ofic 213. Medellin. Colombia; 2 PREVIVA, School of Public Health, University of Antioquia. Calle 62 # 52-57, Ofic 236. Medellin. Colombia; 3 PREVIVA, School of Public Health, University of Antioquia. Calle 62 # 52-57, Ofic 201. Medellin. Colombia

**Keywords:** Violence, victimization, perpetration, aggression, interpersonal violence, violence witnessing, population survey, Medellin, Colombia

## Abstract

**Background:**

The burden of injury from violence and the costs attributable to violence are extremely high in Colombia. Despite a dramatic decline in homicides over the last ten years, homicide rate in Medellin, Colombia second largest city continues to rank among the highest of cities in Latin America. This study aims to estimate the prevalence and distribution of witnesses, victims and perpetrators of different forms of interpersonal violence in a representative sample of the general population in Medellin in 2007.

**Methods:**

A face-to-face survey was carried out on a random selected, non-institutionalized population aged 12 to 60 years, with a response rate of 91% yielding 2,095 interview responses.

**Results:**

We present the rates of prevalence for having been a witness, victim, or perpetrator for different forms of violence standardized using the WHO truncated population pyramid to allow for cross-national comparison. We also present data on verbal aggression, fraud and deception, yelling and heavy pranks, unarmed aggression during last year, and armed threat, other severe threats, robbery, armed physical aggression, and sexual aggression during the lifetime, by age, sex, marital and socioeconomic status, and education. Men reported the highest prevalence of being victims, perpetrators and witnesses in all forms of violence, except for robbery and sexual violence. The number of victims per perpetrator was positively correlated with the severity of the type of violence. The highest victimization proportions over the previous twelve months occurred among minors. Perpetrators are typically young unmarried males from lower socio-economic strata.

**Conclusions:**

Due to very low proportion of victimization report to authorities, periodic surveys should be included in systems for epidemiological monitoring of violence, not only of victimization but also for perpetrators. Victimization information allows quantifying the magnitude of different forms of violence, while data on factors associated with aggression and perpetrators are necessary to estimate risk and protective factors that are essential to sound policies for violence prevention formulation.

## Background

Out of 1.6 million deaths that are caused by violence annually in the world, around 90% occur in developing countries [1]. Compared to developed countries, there is still scant empirical information on the magnitude and composition of interpersonal violence, which represents the most widespread type of violence in the world and has become a serious public health problem [2-4]. For instance, Medellin that is the second largest city in Colombia, with a population of nearly 2.5 million has suffered a severe epidemic of violence during past three decades, reaching a peak of 348 homicides per 100,000 inhabitants in 1991. The rate declined as low as 34 homicides per 100,000 inhabitants in 2005 [5] to increase again to 94.4 in the last two years [6]. Even having such an important and long lasting problem, most of the information on violence in Colombia continues to be based on mortality statistics, police reports, statistics on victims attended by the health care system, or reports to the justice system, all of which may severely underestimate the magnitude of the violence due to inaccurate reporting [7,8].

The economic cost of violence is especially high in Colombia, estimated at 4.3% of the country's gross national product (GNP). Among WHO Member States that reported data on violence, Colombia ranks second in terms of cost of violence after Burundi. It was the country with the highest cost of violence as a percentage of GNP in the Americas, followed by El Salvador (2.0% of GNP) and Venezuela (1.9% of GNP) in the year 2002 [9].

The purpose of this article is to estimate the prevalence and distribution of witnesses, victims, and perpetrators of different forms of interpersonal violence in a representative sample of the general population in Medellin in 2007.

## Methods

This cross-sectional survey was carried out in a random sample of non-institutionalized population (12 to 60 years of age) in the urban area of Medellin. The target sample size was 2,300. The actual sample size was 2,095, equivalent to a response rate of 91%. Of the remaining 9%, most did not respond because they were not in the household at the time of the visit. The sample was selected in four stages. First, sampling was conducted proportionate to the population of each of the 16 urban districts (known as *comunas*). Next, street intersections within each district (*comuna*) were selected randomly. Then, a map of properties was used to undertake a census of inhabitants in the first ten to twelve housing units in a clockwise direction in each of the sampling sites selected. In this way, at least 45 persons between 12 and 60 years of age for each site were identified. Finally, twelve (12) persons for each sampling segment were chosen at random among them.

All interviews were conducted by professionals or advanced university students who had undergone previous training. Each face to face interview lasted forty-five minutes to an hour, and was done through personal interviews in the interviewee's home in a place where confidentiality was provided. Interviewees were explained the purpose of the study, its benefits for the community, the aims of the sponsoring and executing institutions of the survey, and interviewees' rights with respect to answering questions and the confidentiality of information. Questions concerning violence were left to the end of the interview, after having established a positive relationship with the participant.

The survey questionnaire was based on a questionnaire that was previously used in Bogota, Colombia [3], Itagui,[10] and in the Medellin metropolitan area, [2,4] and was based on a set of existing measurement forms [11-13]. The original questionnaire was reviewed by four experts who evaluated its content validity and comprehension. It was tested in extreme known groups (e.g., male and female populations in prisons, meditation and religious groups) and only those questions with discriminatory validity were included. A pilot was carried out with 48 families in two neighborhoods of Medellin and with two focal groups to validate the comprehensibility of the instrument.

The research protocol was reviewed and approved by the Ethics Committee of the School of Public Health at the University of Antioquia. Prior, informed consent was obtained using consent forms approved by the School of Public Health Ethics Committee, which were presented and explained to both the interviewer and interviewee before each interview. Prior consent for minors was obtained from the father, mother, or other responsible adult present in the household.

The items that explored violence during the past year were verbal violence, fraud/deception, yelling and heavy pranks, and unarmed aggression. Respondents were asked to report the incidence of these items during the last year, while the more severe items were reported over respondent's lifetime. Severe aggressions included armed threats, severe threats, unarmed robbery, armed physical aggression and sexual aggression. See table 1 for description of each one of these violence forms.

**Table 1:** Types of violence studied.

TYPE OF VIOLENCE	COMPONENTS
Verbal aggression	Insults, angry shouting

Defraud or deceive	Taking advantage of others, fraud, deceit

Yelling and heavy pranks	Yelling and practical jokes

Unarmed aggression	Strike with the hand or fist with an object, such as a stick or belt

Less severe threat	Threats of beating, threats of injuring, threats of hitting with an object,

Armed threat	Threats with a sharp instrument, knife or firearm

Severe threat	Threats of extortion (being coerced to provide money, property, or services), threats of forced displacement

Unarmed robbery	Robbery with no use of a sharp instrument, knife or firearm

Armed physical aggression	To attack with a sharp instrument, knife or firearm

Sexual aggression	Rape, attempted rape, or not consented caresses

Medellin and metropolitan area. 2007.

For each type of violence, interviewees' were questioned about their experience as witnesses, as victims, and as perpetrators. Besides this information, the questionnaire also included information on sociodemographic characteristics. Socioeconomic status was defined using the household classification scheme employed by the municipality, which is based on the physical characteristics of the household and the block where it is located. The questionnaire also addresses other themes, such as psychoactive substance abuse and protective and risk factors related to personal characteristics, family, peers, neighborhood, and society variables. Each research supervisor carried out a daily revision of missing and inconsistent data, and verified the completion of a sample of 10% of the interviews through follow-up with the respondents by telephone.

Rates of prevalence and their respective confidence intervals were calculated and analyzed by age, sex, level of education, and socioeconomic status. Prevalence rates were compared using the Z-test for difference in proportions and the Chi-square test for association or trend, as appropriate. The Chi-square test for trend was used to compare a nominal variable with an ordinal variable. When the value of the test was significant, the value was used. Otherwise, the value of the Chi-square test of association was used.

## Results

A response rate of 91% was obtained. In total, there were 2,095 interviews, implying a rate of sampling error of 2% and a confidence level of 0.95. The sample consisted of 43.4% males, 20.7% minors (less than 18), 13.7% within the age range 19 to 24, and 42.5% from the lower socioeconomic strata. These sample characteristics do not differ from projections based on the latest population census (2005) carried out by National Department of Statistics [14].

In order to allow for cross-country comparison, adjusted prevalence rates were estimated based on the universal population truncated for ages between 15 and 60 [15] (Table 2).

**Table 2:** Adjusted prevalence rates per 100 (95% CI) of violence in Medellin.

AGRESSION TYPES	VICTIM	PERPETRATOR	WITNESS
***DURING PAST YEAR***			

Yelling and heavy pranks	25.0	18.2	49.6
	(23.2 - 26.9)	(16.6 - 20.0)	(47.4 - 51.7)
Insults or angry shouting	31.4	27.4	60.9
	(29.4 - 33.5)	(25.5 - 29.3)	(58.8 - 63.0)

**Total verbal aggression**	**31.4**	**27.4**	**60.9**
	(29.4 - 33.5)	(25.5 - 29.3)	(58.8 - 63.0)

Slapping, hitting with arm or fist	4.1	4.0	19.0
	(3.3 - 5.1)	(3.2 - 4.9)	(17.4 - 20.8)
Hit with an object	3.8	5.8	21.2
	(3.0 - 4.7)	(4.8 - 6.9)	(19.4 - 23.0)

**Total unarmed physical aggression**	**6.8**	**8.7**	**30.4**
	(5.8 - 8.0)	(7.5 - 10.0)	(28.4 - 32.4)

***DURING LIFETIME***			

Beating or injuring threats	9.3	6.6	32.6
	(8.1 - 10.6)	(5.6 - 7.8)	(30.6 - 34.7)
Hitting with an object threats	4.5	6.0	22.8
	(3.6 - 5.4)	(5.0 - 7.1)	(21.0 - 24.7)

**Total threats of unarmed physical aggression**	**11.3**	**10.3**	**39.6**

	(10.0 - 12.8)	(9.0 - 11.6)	(37.5 - 41.7)
To injure with sharp instrument or knife	5.5	1.1	23.1
	(4.6 - 6.6)	(0.7 - 1.7)	(21.3 - 25.0)
To shoot at a person	3.8	1.4	25.6
	(3.0 - 4.7)	(0.9 - 2.0)	(23.7 - 27.5)

**Total armed aggression**	**8.0**	**2.1**	**34.5**

	(6.9 - 9.3)	(1.5 - 2.8)	(32.5 - 36.6)
Rape intent	2.4	0.1	3.5
	(1.8 - 3.2)	(0.0 - 0.4)	(2.8 - 4.4)
Rape	1.3	0.1	1.6
	(0.9 - 1.9)	(0.0 - 0.3)	(1.1 - 2.3)
Non consented caresses	4.8	0.9	10.2
	(4.0 - 5.9)	(0.6 - 1.5)	(9.0 - 11.6)

**Total sexual aggression**	**6.3**	**0.1**	**3.5**

	(5.3 - 7.5)	(0.0 - 0.4)	(2.8 - 4.4)
Extortion threat	5.5	0.6	14.8
	(4.5 - 6.5)	(0.3 - 1.1)	(13.3 - 16.4)
Forced displacement threat	5.2	0.4	15.1
	(4.3 - 6.2)	(0.1 - 0.7)	(13.5 - 16.7)

**Total severe threat**	**9.6**	**0.8**	**24.3**

	(8.4 - 11.0)	(0.5 - 1.3)	(22.5 - 26.2)
Threat with knife or sharp instrument	13.3	1.7	34.9
	(11.9 - 14.9)	(1.2 - 2.3)	(32.9 - 37.0)
Threat with firearm	12.2	1.4	27.0
	(10.8 - 13.6)	(1.0 - 2.0)	(25.1 - 28.9)

**Total armed threat**	**20.0**	**2.6**	**41.4**
	(18.3 - 21.8)	(1.9 - 3.3)	(39.2 - 43.5)

**Robbery with no weapon**	**21.4**	**2.5**	**25.0**
	(19.7 - 23.2)	(1.9 - 3.2)	(23.1 - 26.9)

**Armed assault**	**18.7**	**0.5**	**25.4**
	(17.0 - 20.4)	(0.3 - 1.0)	(23.6 - 27.4)

**To take advantage of others, defraud or deception**	**14.6**	**2.9**	**26.7**
	(13.1 - 16.2)	(2.2 - 3.7)	(24.8 - 28.7)
	n = 2095	n = 2095	n = 2095

Colombia. 2007.

Over the previous year, the most frequent expression of violence was verbal, followed by yelling and heavy pranks, unarmed physical aggression and, representing the lowest proportion, fraud or deception. The highest proportions of violence over the lifespan were unarmed robbery and unarmed threats, followed by armed threats and sexual violence. Participants reported that they were more likely to be a witness to violence than a victim, and more likely to be a victim than a perpetrator. More severe aggression was associated with a higher victim-perpetrator ratio (i.e., the number victims/number perpetrators for each type of violence in the sample). Lesser forms of aggression show 1.0 to 1.2 victims per perpetrator, compared with 10 to 12 in the case of the more severe forms of aggression.

Table 3 contains the total prevalence proportions, and prevalence proportions by age. Although not included in the table, 15.9% of the interviewees stated having witnessed a homicide in their lifetime. Close to 20% had been victims of armed threat or unarmed robbery. Around 8% were victims of severe threats (threat of displacement or murder), and 5% were victims of sexual aggression over their lifetimes.

**Table 3:** Prevalence per 100 (95% CI) of being a victim, perpetrator and witness of different forms of violence by age (12 to 60, both sexes).

Types of aggression	Total	Age groups				P value
		
		12 to 17	18 to 35	36 to 55	56 or more	
**During past year**						

**Verbal aggression**						

Victim	**28.3**	**42.3**	**31.9**	**21.5**	**9.8**	***
	(26.3 - 30.2)	(37.3 - 47.4)	(28.6 - 35.2)	(18.6 - 24.4)	(5.7 - 15.4)	
Perpetrator	**24.3**	**39.2**	**29.3**	**15.8**	**6.1**	***
	(22.5 - 26.2)	(34.3 - 44.3)	(26.1 - 32.5)	(13.2 - 18.4)	(3.0 - 10.9)	
Witness	**59.7**	**71.9**	**65.6**	**52.5**	**37.2**	***
	(57.6 - 61.8)	(67.2 - 76.4)	(62.2 - 69.0)	(48.9 - 56.0)	(29.8 - 45.1)	

**Defraud or deceive**						

Victim	**13.5**	**12.2**	**15.2**	**14.1**	**4.9**	**
	(12.0 - 14.9)	(9.1 - 15.9)	(12.7 - 17.8)	(11.7 - 16.6)	(2.1 - 9.4)	
Perpetrator	**3.0**	**6.0**	**4.1**	**0.8**	**0.6**	***
	(2.2 - 3.7)	(3.8 - 8.8)	(2.7 - 5.5)	(0.2 - 1.4)	(0.0 - 3.4)	
Witness	**26.9**	**37.4**	**29.5**	**21.5**	**15.9**	***
	(25.0 - 28.8)	(32.6 - 42.4)	(26.2 - 32.7)	(18.6 - 24.4)	(10.6 - 22.4)	

**Yelling and heavy pranks**						

Victim	**23.8**	**36.1**	**25.8**	**17.5**	**14.6**	***
	(21.9 - 25.6)	(31.3 - 41.1)	(22.8 - 28.9)	(14.8 - 20.2)	(9.6 - 21.0)	
Perpetrator	**17.1**	**37.4**	**20.2**	**6.9**	**3.7**	***
	(15.5 - 18.7)	(32.6 - 42.4)	(17.3 - 23.0)	(5.1 - 8.6)	(1.4 - 7.8)	
Witness	**47.7**	**68.8**	**53.7**	**36.8**	**21.3**	***
	(45.6 - 49.9)	(63.9 - 73.4)	(50.2 - 57.3)	(33.4 - 40.3)	(15.3 - 28.4)	

**Unarmed aggression**						

Victim	**22.2**	**32.7**	**23.1**	**17.9**	**13.4**	***
	(20.4 - 24.0)	(28.1 - 37.7)	(20.2 - 26.1)	(15.2 - 20.6)	(8.6 - 19.6)	
Perpetrator	**22.2**	**33.2**	**23.5**	**17.5**	**12.8**	***
	(20.5 - 24.0)	(28.6 - 38.2)	(20.5 - 26.5)	(14.8 - 20.2)	(8.1 - 18.9)	
Witness	**29.5**	**43.4**	**29.8**	**24.5**	**19.5**	***
	(27.6 - 31.5)	(38.4 - 48.5)	(26.6 - 33.1)	(21.4 - 27.5)	(13.7 - 26.4)	

**During life time**						

**Armed threat**						

Victim	**20.2**	**8.8**	**23.8**	**23.2**	**15.9**	***
	(18.5 - 21.9)	(6.2 - 12.1)	(20.8 - 26.8)	(20.2 - 26.2)	(10.6 - 22.4)	
Perpetrator	**2.1**	**1.3**	**3.2**	**1.7**	**1.2**	
	(1.5 - 2.8)	(0.4 - 3.0)	(2.0 - 4.5)	(0.8 - 2.6)	(0.1 - 4.3)	
Witness	**39.3**	**35.6**	**45.9**	**36.8**	**29.3**	*
	(37.2 - 41.4)	(30.8 - 40.6)	(42.3 - 49.5)	(33.4 - 40.3)	(22.4 - 36.9)	

**Severe threat**						

Victim	**8.8**	**5.7**	**9.3**	**10.1**	**7.3**	
	(7.6 - 10.0)	(3.6 - 8.5)	(7.3 - 11.3)	(8.0 - 12.2)	(3.8 - 12.4)	
Perpetrator	**0.7**	**0.8**	**0.8**	**0.6**		
	(0.3 - 1.0)	(0.2 - 2.3)	(0.2 - 1.4)	(0.1 - 1.2)	ND	
Witness	**24.9**	**24.2**	**26.6**	**23.9**	**22.6**	
	(23.0 - 26.7)	(20.0 - 28.8)	(23.5 - 29.8)	(20.9 - 26.9)	(16.4 - 29.7)	

**Unarmed robbery**						

Victim	**21.5**	**19.0**	**21.3**	**24.1**	**15.9**	
	(19.7 - 23.2)	(15.2 - 23.2)	(18.4 - 24.2)	(21.1 - 27.1)	(10.6 - 22.4)	
Perpetrator	**2.1**	**4.7**	**2.5**	**1.0**		***
	(1.5 - 2.8)	(2.8 - 7.3)	(1.4 - 3.5)	(0.3 - 1.8)	**ND**	
Witness	**25.5**	**33.3**	**26.6**	**22.3**	**16.5**	***
	(23.6 - 27.3)	(28.6 - 38.3)	(23.5 - 29.7)	(19.3 - 25.2)	(11.1 - 23.0)	

**Armed physical aggression**						

Victim	**7.6**	**3.1**	**8.4**	**9.2**	**6.7**	*
	(6.5 - 8.7)	(1.6 - 5.4)	(6.4 - 10.4)	(7.2 - 11.2)	(3.4 - 11.7)	
Perpetrator	**1.9**	**0.8**	**2.5**	**1.9**	**1.2**	
	(1.3 - 2.4)	(0.2 - 2.3)	(1.4 - 3.5)	(1.0 - 2.9)	(0.1 - 4.3)	
Witness	**33.3**	**27.8**	**37.1**	**34.1**	**24.4**	***
	(31.3 - 35.3)	(23.4 - 32.6)	(33.7 - 40.6)	(30.7 - 37.4)	(18.0 - 31.7)	

**Sexual aggression**						

Victim	**5.9**	**3.9**	**7.0**	**6.2**	**4.3**	
	(4.9 - 6.9)	(2.2 - 6.3)	(5.2 - 8.8)	(4.5 - 7.9)	(1.7 - 8.6)	
Perpetrator	**1.1**	**1.6**	**1.8**	**0.5**	**ND**	*
	(0.7 - 1.6)	(0.6 - 3.4)	(0.9 - 2.7)	(0.0 - 1.0)		
Witness	**11.6**	**14.5**	**13.4**	**10.1**	**3.7**	***
	(10.3 - 13.0)	(11.2 - 18.5)	(11.0 - 15.8)	(8.0 - 12.2)	(1.4 - 7.8)	

	n = 2095	n = 385	n = 774	n = 772	n = 164	

Medellin, Colombia. 2007.

*P < 0.05 ** p < 0.01 *** p < 0.001

NA: Does not apply ND: No data

Younger age groups were more likely to report being victims, perpetrators, and witnesses to verbal aggression, fraud or deception, yelling and heavy pranks, unarmed aggression and unarmed robbery. Younger age groups were also more likely to be witnesses and victims of armed aggression. Minors (18 years of age or less) generally showed the highest incidence of being perpetrators and were more likely to be the victims of the different forms of violence compared to other age groups. Nearly half had already witnessed a robbery, and one-sixth indicated having witnessed at least one sexual aggression. The prevalence of being an armed physical perpetrator and severe threats were similar in all age groups.

Marital status was determined as being without a partner (single, divorced, widowed or widower) or with a partner (married or common law). Participants without a partner were significantly more aggressive in so much as verbal violence, deception/fraud, yelling and heavy pranks, physical aggression without a weapon, robbery, and sexual aggression were concerned, while those who had a partner were more so in terms of unarmed threats. There is a positive association between level of education and being a victim of deception/fraud, armed threats, robbery, and sexual violence. Respondents with a secondary education showed a greater likelihood of being victims of verbal aggression and yelling and heavy pranks, as well as a higher chance of being a victim of armed threats, unarmed robbery and sexual aggression as higher levels of education imply older age. Respondents with secondary educations were more likely to have committed verbal aggression and yelling and heavy pranks, and higher levels of education were also associated with a higher risk of committing aggression by armed threats (Table 4).

**Table 4:** Prevalence proportion per 100 (95% CI) of being the victim, perpetrator or witness of different forms of violence over the previous year and lifespan by marital status and educational level.

Types of violence	Marital status		P value	Educational level				P value
				
	Without partner	With partner		Elementary	High school	Technical school	University or graduate studies	
**During past year**								

**Verbal aggression**								

Victim	**33.0**	**21.0**	***	**22.1**	**31.9**	**26.0**	**25.6**	***
	(30.4 - 35.6)	(18.3 - 23.8)		(18.1 - 26.6)	(29.2 - 34.7)	(20.1 - 32.6)	(21.3 - 30.3)	
Perpetrator	**29.2**	**16.9**	***	**18.8**	**28.4**	**21.6**	**20.3**	***
	(26.7 - 31.7)	(14.4 - 19.5)		(15.0 - 23.0)	(25.8 - 31.1)	(16.1 - 27.9)	(16.4 - 24.7)	
Witness	**64.8**	**51.9**	***	**47.4**	**62.1**	**59.3**	**66.0**	***
	(62.1 - 67.5)	(48.5 - 55.4)		(42.3 - 52.5)	(59.2 - 65.0)	(52.2 - 66.1)	(61.0 - 70.7)	

**Defraud or deceive**								

Victim	**15.5**	**10.5**	***	**11.5**	**12.4**	**15.7**	**17.7**	**
	(13.5 - 17.4)	(8.4 - 12.5)		(8.5 - 15.1)	(10.4 - 14.3)	(11.0 - 21.4)	(14.0 - 21.9)	
Perpetrator	**4.3**	**1.0**	***	**1.0**	**4.0**	**1.5**	**2.9**	*
	(3.2 - 5.4)	(0.3 - 1.6)		(0.3 - 2.6)	(2.8 - 5.1)	(0.3 - 4.2)	(1.5 - 5.1)	
Witness	**30.7**	**21.0**	***	**17.2**	**29.2**	**22.5**	**33.0**	***
	(28.2 - 33.3)	(18.3 - 23.8)		(13.5 - 21.3)	(26.5 - 31.8)	(17.0 - 28.9)	(28.3 - 38.0)	

**Yelling and heavy pranks**								

Victim	**27.9**	**17.4**	***	**18.5**	**26.9**	**18.1**	**23.0**	**
	(25.4 - 30.4)	(14.9 - 20.0)		(14.7 - 22.7)	(24.3 - 29.5)	(13.1 - 24.1)	(18.8 - 27.5)	
Perpetrator	**22.6**	**8.8**	***	**9.9**	**22.4**	**12.3**	**12.4**	***
	(20.3 - 24.9)	(6.9 - 10.7)		(7.1 - 13.3)	(19.9 - 24.8)	(8.1 - 17.6)	(9.3 - 16.1)	
Witness	**55.2**	**36.5**	***	**33.6**	**52.2**	**45.1**	**52.0**	***
	(52.4 - 57.9)	(33.3 - 39.8)		(28.9 - 38.6)	(49.2 - 55.1)	(38.1 - 52.2)	(46.8 - 57.1)	

**Unarmed aggression**								

Victim	**24.8**	**18.3**	***	**21.1**	**23.6**	**21.6**	**20.3**	
	(22.4 - 27.2)	(15.6 - 20.9)		(17.1 - 25.5)	(21.1 - 26.1)	(16.1 - 27.9)	(16.4 - 24.7)	
Perpetrator	**24.5**	**18.9**	***	**21.1**	**23.5**	**23.0**	**20.3**	
	(22.1 - 26.9)	(16.2 - 21.5)		(17.1 - 25.5)	(21.0 - 26.0)	(17.4 - 29.4)	(16.4 - 24.7)	
Witness	**32.8**	**24.6**	***	**29.2**	**31.2**	**26.0**	**27.7**	
	(30.2 - 35.4)	(21.7 - 27.6)		(24.7 - 34.0)	(28.5 - 34.0)	(20.1 - 32.6)	(23.3 - 32.5)	

**During life time**								

**Armed threat**								

Victim	**20.1**	**20.3**	***	**16.7**	**17.1**	**26.0**	**30.1**	***
	(17.9 - 22.3)	(17.6 - 23.0)		(13.1 - 20.8)	(14.8 - 19.3)	(20.1 - 32.6)	(25.5 - 35.0)	
Perpetrator	**2.0**	**2.4**	**	**2.3**	**2.9**	**0.5**	**0.8**	*
	(1.2 - 2.7)	(1.4 - 3.4)		(1.1 - 4.4)	(1.9 - 3.9)	(0.0 - 2.7)	(0.2 - 2.3)	
Witness	**40.5**	**37.6**	***	**31.8**	**38.7**	**45.6**	**45.9**	***
	(37.8 - 43.2)	(34.3 - 40.9)		(27.1 - 36.7)	(35.9 - 41.6)	(38.6 - 52.7)	(40.8 - 51.1)	

**Severe threat**								

Victim	**8.1**	**9.7**	***	**0.8**	**0.8**	**0.5**	**0.3**	
	(6.6 - 9.6)	(7.7 - 11.7)		(0.2 - 2.3)	(0.3 - 1.3)	(0.0 - 2.7)	(0.0 - 1.5)	
Perpetrator	**0.8**	**0.5**		**23.0**	**24.2**	**26.0**	**28.0**	
	(0.3 - 1.3)	(0.0 - 1.0)		(18.9 - 27.5)	(21.6 - 26.7)	(20.1 - 32.6)	(23.5 - 32.8)	
Witness	**25.4**	**24.1**	***	**10.2**	**7.7**	**9.8**	**9.0**	
	(23.0 - 27.8)	(21.2 - 27.0)		(7.3 - 13.6)	(6.1 - 9.2)	(6.1 - 14.7)	(6.3 - 12.3)	

**Unarmed robbery**								

Victim	**21.6**	**21.4**	***	**16.9**	**20.2**	**27.5**	**26.9**	***
	(19.3 - 23.8)	(18.6 - 24.2)		(13.3 - 21.1)	(17.9 - 22.6)	(21.5 - 34.1)	(22.5 - 31.7)	
Perpetrator	**2.8**	**1.2**	***	**2.3**	**3.0**	**1.0**	**0.3**	**
	(1.9 - 3.7)	(0.5 - 1.9)		(1.1 - 4.4)	(2.0 - 4.0)	(0.1 - 3.5)	(0.0 - 1.5)	
Witness	**27.9**	**21.8**	***	**18.2**	**27.1**	**25.0**	**29.0**	**
	(25.4 - 30.4)	(19.0 - 24.6)		(14.5 - 22.5)	(24.5 - 29.7)	(19.2 - 31.5)	(24.5 - 33.9)	

**Armed physical aggression**								

Victim	**7.2**	**8.2**	***	**9.9**	**6.5**	**8.3**	**7.7**	
	(5.8 - 8.6)	(6.3 - 10.0)		(7.1 - 13.3)	(5.0 - 7.9)	(4.9 - 13.0)	(5.2 - 10.8)	
Perpetrator	**1.9**	**1.8**	*	**2.6**	**2.0**	**1.5**	**1.1**	
	(1.1 - 2.7)	(0.9 - 2.7)		(1.3 - 4.7)	(1.2 - 2.8)	(0.3 - 4.2)	(0.3 - 2.7)	
Witness	**33.0**	**33.5**	***	**28.4**	**32.8**	**36.3**	**38.3**	**
	(30.4 - 35.6)	(30.3 - 36.7)		(23.9 - 33.2)	(30.0 - 35.5)	(29.7 - 43.3)	(33.3 - 43.4)	

**Sexual aggression**								

Victim	**7.0**	**4.3**	***	**4.9**	**4.5**	**8.3**	**9.5**	***
	(5.6 - 8.4)	(2.9 - 5.7)		(3.0 - 7.6)	(3.3 - 5.7)	(4.9 - 13.0)	(6.7 - 12.9)	
Perpetrator	**1.7**	**0.4**	***	**0.5**	**1.4**	**0.5**	**1.6**	
	(1.0 - 2.4)	(0.0 - 0.8)		(0.1 - 1.9)	(0.7 - 2.0)	(0.0 - 2.7)	(0.6 - 3.4)	
Witness	**13.4**	**9.0**	***	**8.6**	**11.4**	**12.7**	**15.3**	**
	(11.5 - 15.3)	(7.1 - 11.0)		(6.0 - 11.9)	(9.5 - 13.2)	(8.5 - 18.1)	(11.8 - 19.3)	
	n = 1262	n = 832		n = 384	n = 1108	n = 204	n = 379	

Population 12 - 60 years of age. Medellin, Colombia. 2007.

*P < 0.05 ** p < 0.01 *** p < 0.001

NA: Do not apply

ND: No data

Table 5 details the level of violence according to employment status over the previous year and socioeconomic stratum (SES). Those who were employed during each of the twelve months were less likely to have been perpetrators or victims of verbal violence, fraud/deception, yelling and heavy pranks, and unarmed aggression. Participants that had been unemployed for 4 to 8 months were in general more likely to be perpetrators and victims of violence than those who had experienced the same situation for less than 4 months or more than 8; however, these differences were not significant.

**Table 5:** Proportion of prevalence per 100 (95% CI) of being a victim, perpetrator and witness of different forms of violence over the previous year and lifespan, by employment status over the previous year and SES.

Types of violence	Unemployment, past year				P value	Socioeconomic status			P value
				
	0 months	1 - 3 months	4 - 8 months	9 - 12 months		Low	Middle	High	
**During past year**									

**Verbal aggression**									

Victim	**14.1**	**36.2**	**45.7**	**32.7**	***	**29.4**	**27.9**	**25.5**	
	(10.4 - 18.5)	(25.0 - 48.7)	(34.6 - 57.1)	(26.6 - 39.4)		(26.4 - 32.4)	(25.0 - 30.7)	(20.2 - 31.4)	
Perpetrator	**11.1**	**30.4**	**44.4**	**24.5**	***	**26.6**	**23.6**	**19.1**	*
	(7.8 - 15.2)	(19.9 - 42.7)	(33.4 - 55.9)	(19.0 - 30.8)		(23.7 - 29.5)	(20.9 - 26.3)	(14.4 - 24.5)	
Witness	**44.1**	**68.1**	**74.1**	**66.8**	***	**59.4**	**58.6**	**64.9**	
	(38.5 - 49.9)	(55.8 - 78.8)	(63.1 - 83.2)	(60.2 - 73.0)		(56.1 - 62.7)	(55.4 - 61.7)	(58.7 - 70.8)	

**Defraud or deceive**									

Victim	**9.5**	**17.4**	**22.2**	**14.5**	*	**12.9**	**13.8**	**13.9**	
	(6.4 - 13.3)	(9.3 - 28.4)	(13.7 - 32.8)	(10.2 - 19.9)		(10.7 - 15.1)	(11.6 - 16.0)	(9.9 - 18.9)	
Perpetrator	**1.6**	**4.3**	**3.7**	**5.0**		**3.4**	**3.1**	**0.8**	
	(0.5 - 3.8)	(0.9 - 12.2)	(0.8 - 10.4)	(2.5 - 8.8)		(2.2 - 4.6)	(2.0 - 4.3)	(0.1 - 2.8)	
Witness	**18.6**	**39.1**	**25.9**	**27.3**	**	**28.5**	**24.5**	**30.3**	
	(14.4 - 23.4)	(27.6 - 51.6)	(16.8 - 36.9)	(21.5 - 33.7)		(25.6 - 31.5)	(21.8 - 27.3)	(24.7 - 36.4)	

**Yelling and heavy pranks**									

Victim	**15.7**	**27.5**	**24.7**	**25.9**	*	**25.1**	**23.4**	**20.7**	
	(11.8 - 20.3)	(17.5 - 39.6)	(15.8 - 35.5)	(20.3 - 32.2)		(22.2 - 27.9)	(20.7 - 26.1)	(15.9 - 26.3)	
Perpetrator	**9.5**	**13.0**	**19.8**	**18.2**	*	**19.6**	**16.2**	**12.0**	
	(6.4 - 13.3)	(6.1 - 23.3)	(11.7 - 30.1)	(13.3 - 23.9)		(16.9 - 22.2)	(13.9 - 18.6)	(8.2 - 16.6)	
Witness	**39.9**	**50.7**	**53.1**	**44.5**		**47.9**	**47.9**	**46.6**	
	(34.3 - 45.6)	(38.4 - 63.0)	(41.7 - 64.3)	(37.9 - 51.4)		(44.5 - 51.2)	(44.7 - 51.1)	(40.3 - 53.0)	

**Unarmed aggression**									

Victim	**16.0**	**29.0**	**35.8**	**23.6**	***	**25.6**	**20.2**	**17.5**	***
	(12.1 - 20.6)	(18.7 - 41.2)	(25.4 - 47.2)	(18.2 - 29.8)		(22.8 - 28.5)	(17.7 - 22.8)	(13.0 - 22.8)	
Perpetrator	**15.4**	**30.4**	**33.3**	**23.2**	***	**25.1**	**21.3**	**15.9**	**
	(11.5 - 19.9)	(19.9 - 42.7)	(23.2 - 44.7)	(17.8 - 29.3)		(22.2 - 27.9)	(18.7 - 23.9)	(11.6 - 21.1)	
Witness	**20.9**	**42.0**	**43.2**	**28.6**	***	**33.4**	**26.3**	**28.3**	**
	(16.5 - 25.9)	(30.2 - 54.5)	(32.2 - 54.7)	(22.8 - 35.1)		(30.3 - 36.5)	(23.5 - 29.1)	(22.8 - 34.3)	

**During life time**									

**Armed threat**									

Victim	**19.9**	**31.9**	**23.5**	**20.9**		**17.5**	**22.1**	**22.3**	*
	(15.6 - 24.9)	(21.2 - 44.2)	(14.8 - 34.2)	(15.7 - 26.9)		(15.0 - 20.0)	(19.5 - 24.8)	(17.3 - 28.0)	
Perpetrator	**1.3**	**7.2**	**4.9**	**0.9**	**	**2.8**	**2.0**	**0.4**	*
	(0.4 - 3.3)	(2.4 - 16.1)	(1.4 - 12.2)	(0.1 - 3.2)		(1.7 - 3.9)	(1.1 - 2.9)	(0.0 - 2.2)	
Witness	**31.7**	**49.3**	**49.4**	**44.1**	**	**38.3**	**40.0**	**40.2**	
	(26.5 - 37.2)	(37.0 - 61.6)	(38.1 - 60.7)	(37.4 - 50.9)		(35.1 - 41.5)	(36.9 - 43.2)	(34.1 - 46.6)	

**Severe threat**									

Victim	**9.5**	**8.7**	**6.2**	**12.3**		**0.8**	**0.6**	**0.4**	
	(6.4 - 13.3)	(3.3 - 18.0)	(2.0 - 13.8)	(8.2 - 17.4)		(0.2 - 1.4)	(0.1 - 1.1)	(0.0 - 2.2)	
Perpetrator	**1.0**	**SD**	**SD**	**0.9**		**27.6**	**22.9**	**22.7**	*
	(0.2 - 2.8)			(0.1 - 3.2)		(24.7 - 30.5)	(20.2 - 25.6)	(17.7 - 28.4)	
Witness	**20.0**	**30.4**	**35.8**	**30.5**	**	**9.8**	**7.9**	**8.8**	
	(15.7 - 24.9)	(19.9 - 42.7)	(25.4 - 47.2)	(24.4 - 37.0)		(7.8 - 11.7)	(6.2 - 9.6)	(5.6 - 13.0)	

**Unarmed robbery**									

Victim	**12.7**	**30.4**	**19.8**	**23.6**	***	**19.7**	**21.6**	**27.5**	*
	(9.2 - 17.0)	(19.9 - 42.7)	(11.7 - 30.1)	(18.2 - 29.8)		(17.1 - 22.3)	(19.0 - 24.2)	(22.1 - 33.5)	
Perpetrator	**2.0**	**2.9**	**4.9**	**1.8**		**3.1**	**1.7**	**0.4**	**
	(0.7 - 4.2)	(0.4 - 10.1)	(1.4 - 12.2)	(0.5 - 4.6)		(2.0 - 4.3)	(0.9 - 2.5)	(0.0 - 2.2)	
Witness	**19.6**	**36.2**	**25.9**	**28.2**	**	**24.1**	**26.8**	**25.1**	
	(15.3 - 24.5)	(25.0 - 48.7)	(16.8 - 36.9)	(22.3 - 34.6)		(21.3 - 26.9)	(24.0 - 29.6)	(19.9 - 30.9)	

**Armed physical aggression**									

Victim	**7.2**	**13**	**13.6**	**9.1**		**8.3**	**7.4**	**5.6**	
	(4.6 - 10.7)	(6.1 - 23.3)	(7.0 - 23.0)	(5.6 - 13.7)		(6.5 - 10.1)	(5.8 - 9.1)	(3.1 - 9.2)	
Perpetrator	**1.3**	**4.3**	**2.5**	**3.6**		**2.5**	**1.7**	**0.4**	*
	(0.4 - 3.3)	(0.9 - 12.2)	(0.3 - 8.6)	(1.6 - 7.0)		(1.5 - 3.5)	(0.9 - 2.5)	(0.0 - 2.2)	
Witness	**27.1**	**39.1**	**42.0**	**41.8**	**	**34.7**	**33.0**	**29.1**	
	(22.2 - 32.5)	(27.6 - 51.6)	(31.1 - 53.5)	(35.2 - 48.6)		(31.6 - 37.8)	(30.0 - 36.0)	(23.5 - 35.1)	

**Sexual aggression**									

Victim	**2.6**	**11.6**	**9.9**	**5.0**	**	**4.7**	**6.6**	**7.6**	*
	(1.1 - 5.1)	(5.1 - 21.6)	(4.4 - 18.5)	(2.5 - 8.8)		(3.3 - 6.1)	(5.0 - 8.2)	(4.6 - 11.6)	
Perpetrator	**0.7**	**4.3**	**2.5**	**1.4**		**1.2**	**1.3**	**0.4**	
	(0.1 - 2.3)	(0.9 - 12.2)	(0.3 - 8.6)	(0.3 - 3.9)		(0.5 - 2.0)	(0.6 - 2.0)	(0.0 - 2.2)	
Witness	**5.9**	**14.5**	**14.8**	**11.4**	*	**11.0**	**11.5**	**14.3**	
	(3.5 - 9.1)	(7.2 - 25.0)	(7.9 - 24.4)	(7.5 - 16.3)		(9.0 - 13.1)	(9.5 - 13.6)	(10.3 - 19.3)	
	n = 306	n = 69	n = 81	n = 220		n = 890	n = 954	N = 251	

Population 12 - 60 years of age. Medellin, Colombia. 2007.

*P < 0.05 ** p < 0.01 *** p < 0.001

NA: Do not apply

ND: No data

Lower socioeconomic status was associated with a higher likelihood of being a verbal perpetrator, yelling and heavy pranks, unarmed aggression, severe threats, unarmed robbery and armed physical aggression. It was found that, the higher a respondent's socioeconomic status, the greater was their likelihood of being a victim of verbal aggression and armed threat.

Bivariate analysis by sex revealed that men were the main victims of violence, except in cases of unarmed robbery and sexual violence. There was difference between sexes of having witnessed violence or having been perpetrators; they were higher in men for all forms of violence studied (Table 6).

**Table 6:** Prevalence per 100 (95% CI) of being a victim, perpetrator and witness of different forms of violence, by sex and marital status.

Types of violence	Sex		P value	Men		P value	Women		P value
						
	Man	Woman		Without partner	With partner		Without partner	With partner	
**During past year**									

**Verbal aggression**									

Victim	**31.8**	**25.6**	**	**37.4**	**23.0**	***	**29.6**	**19.6**	***
	(28.7 - 34.8)	(23.1 - 28.1)		(33.4 - 41.6)	(18.7 - 27.7)		(26.3 - 33.1)	(16.1 - 23.4)	
Perpetrator	**28.1**	**21.4**	***	**34.7**	**17.9**	***	**25.0**	**16.2**	***
	(25.2 - 31.1)	(19.1 - 23.8)		(30.8 - 38.9)	(14.1 - 22.3)		(21.8 - 28.2)	(13.0 - 19.8)	
Witness	**63.8**	**56.5**	***	**70.0**	**54.3**	***	**60.8**	**50.1**	***
	(60.6 - 67.0)	(53.7 - 59.4)		(66.0 - 73.8)	(49.0 - 59.6)		(57.1 - 64.4)	(45.5 - 54.7)	

**Defraud or deceive**									

Victim	**17.5**	**10.4**	***	**19.7**	**14.0**	*	**12.1**	**7.8**	*
	(15.0 - 19.9)	(8.6 - 12.1)		(16.5 - 23.3)	(10.6 - 18.0)		(9.7 - 14.5)	(5.5 - 10.6)	
Perpetrator	**4.5**	**1.8**	***	**6.1**	**2.0**	***	**2.8**	**0.2**	***
	(3.2 - 5.9)	(1.0 - 2.5)		(4.1 - 8.2)	(0.8 - 4.0)		(1.6 - 4.0)	(0.0 - 1.2)	
Witness	**35.1**	**20.7**	***	**40.0**	**27.5**	***	**23.6**	**16.2**	**
	(32.0 - 38.2)	(18.4 - 23.0)		(35.9 - 44.2)	(22.9 - 32.4)		(20.4 - 26.7)	(13.0 - 19.8)	

**Yelling and heavy pranks**									

Victim	**29.9**	**19.1**	***	**34.5**	**22.7**	***	**22.7**	**13.5**	***
	(26.9 - 32.9)	(16.8 - 21.3)		(30.6 - 38.7)	(18.4 - 27.4)		(19.6 - 25.8)	(10.5 - 16.9)	
Perpetrator	**26.0**	**10.3**	***	**33.5**	**14.6**	***	**14.1**	**4.4**	***
	(23.2 - 28.9)	(8.6 - 12.0)		(29.5 - 37.6)	(11.1 - 18.7)		(11.5 - 16.7)	(2.8 - 6.7)	
Witness	**54.6**	**42.4**	***	**64.0**	**40.1**	***	**48.2**	**33.9**	***
	(51.3 - 57.9)	(39.6 - 45.3)		(59.9 - 68.0)	(34.9 - 45.3)		(44.5 - 52.0)	(29.6 - 38.3)	

**Unarmed aggression**									

Victim	**26.3**	**19.1**	***	**30.2**	**20.2**	***	**20.6**	**16.8**	
	(23.4 - 29.1)	(16.8 - 21.3)		(26.4 - 34.2)	(16.1 - 24.7)		(17.6 - 23.6)	(13.6 - 20.5)	
Perpetrator	**25.2**	**20.0**	**	**28.4**	**20.2**	***	**21.4**	**17.9**	
	(22.3 - 28.0)	(17.7 - 22.3)		(24.7 - 32.3)	(16.1 - 24.7)		(18.4 - 24.5)	(14.6 - 21.6)	
Witness	**34.6**	**25.7**	***	**39.1**	**27.7**	***	**27.9**	**22.3**	*
	(31.5 - 37.7)	(23.2 - 28.1)		(35.0 - 43.3)	(23.1 - 32.7)		(24.6 - 31.2)	(18.6 - 26.3)	

**During life time**									

**Armed threat**									

Victim	**27.3**	**14.8**	***	**26.9**	**27.7**		**14.8**	**14.7**	
	(24.4 - 30.1)	(12.7 - 16.8)		(23.3 - 30.8)	(23.1 - 32.7)		(12.2 - 17.4)	(11.7 - 18.2)	
Perpetrator	**4.1**	**0.7**	***	**3.6**	**4.8**		**0.7**	**0.6**	
	(2.8 - 5.3)	(0.2 - 1.1)		(2.1 - 5.2)	(2.8 - 7.5)		(0.1 - 1.3)	(0.1 - 1.8)	
Witness	**46.5**	**33.8**	***	**49.7**	**41.5**	*	**33.3**	**34.7**	
	(43.2 - 49.8)	(31.1 - 36.5)		(45.5 - 54.0)	(36.3 - 46.8)		(29.8 - 36.9)	(30.5 - 39.2)	

**Severe threat**									

Victim	**10.2**	**7.7**	*	**9.4**	**11.5**		**7.1**	**8.4**	
	(8.3 - 12.2)	(6.2 - 9.2)		(7.0 - 11.8)	(8.4 - 15.3)		(5.2 - 8.9)	(6.1 - 11.3)	
Perpetrator	**1.1**	**0.3**	*	**1.3**	**0.8**		**0.4**	**0.2**	
	(0.4 - 1.8)	(0.0 - 0.7)		(0.3 - 2.2)	(0.2 - 2.4)		(-0.1 - 0.9)	(0.0 - 1.2)	
Witness	**31.0**	**20.2**	***	**32.2**	**29.1**		**20.1**	**20.3**	
	(28.0 - 34.0)	(17.9 - 22.4)		(28.3 - 36.3)	(24.5 - 34.1)		(17.1 - 23.0)	(16.8 - 24.2)	

**Unarmed robbery**									

Victim	**22.1**	**21.0**		**22.2**	**21.8**		**21.0**	**21.1**	
	(19.4 - 24.8)	(18.7 - 23.3)		(18.8 - 25.9)	(17.7 - 26.5)		(18.0 - 24.0)	(17.5 - 25.0)	
Perpetrator	**4.2**	**0.6**	***	**5.4**	**2.2**	**	**0.7**	**0.4**	
	(2.9 - 5.5)	(0.2 - 1.0)		(3.5 - 7.3)	(1.0 - 4.4)		(0.1 - 1.3)	(0.1 - 1.5)	
Witness	**30.5**	**21.6**	***	**35.0**	**23.5**	***	**22.4**	**20.4**	
	(27.5 - 33.5)	(19.3 - 23.9)		(31.0 - 39.1)	(19.2 - 28.3)		(19.4 - 25.5)	(16.9 - 24.3)	

**Armed physical aggression**									

Victim	**12.5**	**3.8**	***	**11.0**	**14.8**		**4.2**	**3.2**	
	(10.4 - 14.7)	(2.7 - 4.9)		(8.5 - 13.9)	(11.3 - 19.0)		(2.7 - 5.7)	(1.8 - 5.2)	
Perpetrator	**3.7**	**0.4**	***	**3.6**	**3.9**		**0.6**	**0.2**	
	(2.5 - 5.0)	(0.1 - 0.8)		(2.1 - 5.2)	(2.2 - 6.5)		(0.0 - 1.1)	(0.0 - 1.2)	
Witness	**42.1**	**26.5**	***	**43.0**	**40.6**		**25.2**	**28.2**	
	(38.9 - 45.3)	(24.0 - 29.0)		(38.9 - 47.3)	(35.5 - 45.9)		(22.0 - 28.4)	(24.2 - 32.5)	

**Sexual aggression**									

Victim	**3.1**	**8.1**	***	**4.0**	**1.7**	*	**9.3**	**6.3**	
	(2.0 - 4.2)	(6.5 - 9.7)		(2.3 - 5.6)	(0.6 - 3.6)		(7.2 - 11.4)	(4.3 - 8.9)	
Perpetrator	**2.3**	**0.3**	***	**3.3**	**0.8**	**	**0.4**	**ND**	NA
	(1.3 - 3.3)	(0.0 - 0.5)		(1.8 - 4.7)	(0.2 - 2.4)		(-0.1 - 0.9)		
Witness	**15.7**	**8.5**	***	**19.2**	**10.4**	***	**8.9**	**8.0**	
	(13.3 - 18.1)	(6.9 - 10.1)		(16.0 - 22.7)	(7.4 - 14.0)		(6.8 - 11.0)	(5.7 - 10.8)	
	n = 910	n = 1185		n = 553	n = 357		n = 709	n = 475	

Medellin, Colombia. 2003 - 2004

*P < 0.05 ** p < 0.01 *** p < 0.001

ND: No data

NA: Does not apply

The prevalence of victimization and aggression by verbal violence, fraud/deception, yelling and heavy pranks, and sexual aggression was greater among those in a partnership, regardless of the sex of the respondent. Men were more likely than women to be unarmed perpetrators and victims of unarmed aggression. We found no differences by sex between those currently in a partnership and those not with respect to reporting having been a victim or perpetrator of threats with or without a weapon, robbery, and armed aggression (Table 6).

We found no significant differences in yelling and heavy pranks, severe threats, robbery, armed aggression and sexual violence by socio economic status (SES) among both men and women. The proportion of women perpetrators by fraud/deception and verbal aggression decreases as SES increases. Among men, a direct relationship was observed between being a victim of fraud/deception and SES, and an inverse relationship between being a victim of unarmed violence and SES. There were no significant differences in incidence of sexual aggression and sexual victimization by SES independent of sex (Table 7).

**Table 7:** Prevalence per 100 (95% CI) of being a victim, perpetrator and witness of different forms of violence, by socioeconomic status and sex (12 to 60 years of age).

Types of violence	Men			P value	Women			P value
				
	Low	Middle	High		Low	Middle	High	
**During past year**								

**Verbal aggression**								

Victim	**30.4**	**33.5**	**30.0**		**28.6**	**23.6**	**23.0**	
	(26.0 - 35.1)	(28.9 - 38.3)	(20.8 - 40.6)		(24.6 - 32.9)	(20.1 - 27.4)	(16.7 - 30.3)	
Perpetrator	**28.2**	**27.2**	**32.2**		**25.3**	**20.8**	**11.8**	**
	(23.9 - 32.8)	(22.9 - 31.8)	(22.8 - 42.9)		(21.5 - 29.4)	(17.5 - 24.5)	(7.3 - 17.8)	
Witness	**61.0**	**64.1**	**75.6**	*	**58.1**	**54.4**	**59.0**	
	(56.1 - 65.8)	(59.2 - 68.7)	(65.4 - 84.0)		(53.5 - 62.5)	(50.1 - 58.7)	(51.0 - 66.7)	

**Defraud or deceive**								

Victim	**13.7**	**20.4**	**21.1**	*	**12.2**	**8.9**	**9.9**	
	(10.5 - 17.5)	(16.6 - 24.6)	(13.2 - 31.0)		(9.5 - 15.5)	(6.6 - 11.6)	(5.8 - 15.6)	
Perpetrator	**3.7**	**6.1**	**1.1**		**3.1**	**0.9**	**0.6**	*
	(2.1 - 6.0)	(4.0 - 8.8)	(0.0 - 6.0)		(1.8 - 5.1)	(0.1 - 1.7)	(0.0 - 3.4)	
Witness	**33.8**	**34.2**	**44.4**		**24.1**	**17.2**	**22.4**	*
	(29.2 - 38.6)	(29.6 - 39.0)	(34.0 - 55.3)		(20.3 - 28.1)	(14.1 - 20.6)	(16.2 - 29.6)	

**Yelling and heavy pranks**								

Victim	**29.7**	**29.9**	**31.1**		**21.2**	**18.5**	**14.9**	
	(25.3 - 34.3)	(25.5 - 34.5)	(21.8 - 41.7)		(17.6 - 25.1)	(15.3 - 22.0)	(9.8 - 21.4)	
Perpetrator	**28.7**	**24.3**	**22.2**		**11.8**	**10.1**	**6.2**	
	(24.3 - 33.3)	(20.2 - 28.7)	(14.1 - 32.2)		(9.1 - 15.0)	(7.7 - 13.0)	(3.0 - 11.1)	
Witness	**52.7**	**55.1**	**61.1**		**43.8**	**42.4**	**38.5**	
	(47.7 - 57.6)	(50.2 - 60.0)	(50.3 - 71.2)		(39.3 - 48.3)	(38.2 - 46.7)	(31.0 - 46.5)	

**Unarmed aggression**								

Victim	**30.1**	**24.0**	**18.9**		**21.8**	**17.3**	**16.8**	
	(25.7 - 34.9)	(20.0 - 28.5)	(11.4 - 28.5)		(18.2 - 25.7)	(14.2 - 20.8)	(11.4 - 23.5)	
Perpetrator	**27.9**	**23.8**	**18.9**		**22.6**	**19.4**	**14.3**	
	(23.6 - 32.6)	(19.8 - 28.2)	(11.4 - 28.5)		(19.0 - 26.6)	(16.1 - 23.0)	(9.3 - 20.7)	
Witness	**37.7**	**31.6**	**34.4**		**29.7**	**22.3**	**24.8**	*
	(33.0 - 42.6)	(27.1 - 36.3)	(24.7 - 45.2)		(25.6 - 34.0)	(18.9 - 26.1)	(18.4 - 32.3)	

**During life time**								

**Armed threat**								

Victim	**23.5**	**30.3**	**30.0**		**12.4**	**15.9**	**18.0**	
	(19.5 - 28.0)	(25.9 - 35.0)	(20.8 - 40.6)		(9.6 - 15.7)	(12.9 - 19.2)	(12.4 - 24.8)	
Perpetrator	**4.2**	**4.6**	**1.1**		**1.7**	**ND**	**ND**	NA
	(2.4 - 6.6)	(2.8 - 7.1)	(0.0 - 6.0)		(0.7 - 3.2)			
Witness	**43.1**	**48.3**	**53.3**		**34.2**	**33.8**	**32.9**	
	(38.3 - 48.1)	(43.4 - 53.2)	(42.5 - 63.9)		(30.0 - 38.7)	(29.8 - 37.9)	(25.7 - 40.8)	

**Severe threat**								

Victim	**12.0**	**9.2**	**6.7**		**7.9**	**6.8**	**9.9**	
	(9.0 - 15.6)	(6.6 - 12.4)	(2.5 - 13.9)		(5.6 - 10.7)	(4.7 - 8.9)	(5.8 - 15.6)	
Perpetrator	**1.5**	**1.0**			**0.2**	**0.4**	**0.6**	
	(0.5 - 3.2)	(0.3 - 2.5)	ND		(0.0 - 1.2)	(-0.1 - 0.9)	(0.0 - 3.4)	
Witness	**31.9**	**30.6**	**28.9**		**24.0**	**17.0**	**19.3**	*
	(27.4 - 36.6)	(26.2 - 35.3)	(19.8 - 39.4)		(20.2 - 28.0)	(14.0 - 20.5)	(13.5 - 26.2)	

**Unarmed robbery**								

Victim	**19.1**	**23.8**	**27.8**		**20.1**	**19.9**	**27.3**	
	(15.4 - 23.3)	(19.8 - 28.2)	(18.9 - 38.2)		(16.6 - 24.0)	(16.6 - 23.5)	(20.6 - 34.9)	
Perpetrator	**5.1**	**3.9**	**1.1**		**1.5**			NA
	(3.2 - 7.8)	(2.2 - 6.2)	(0.0 - 6.0)		(0.6 - 3.0)	ND	ND	
Witness	**26.0**	**35.0**	**30.0**		**22.4**	**20.7**	**22.4**	
	(21.8 - 30.6)	(30.3 - 39.8)	(20.8 - 40.6)		(18.8 - 26.4)	(17.3 - 24.3)	(16.2 - 29.6)	

**Armed physical aggression**								

Victim	**12.5**	**13.3**	**8.9**		**4.8**	**3.0**	**3.7**	
	(9.5 - 16.1)	(10.2 - 17.0)	(3.9 - 16.8)		(3.0 - 7.1)	(1.5 - 4.4)	(1.4 - 7.9)	
Perpetrator	**4.2**	**3.9**	**1.1**		**1.0**			NA
	(2.4 - 6.6)	(2.2 - 6.2)	(0.0 - 6.0)		(0.3 - 2.4)	ND	ND	
Witness	**42.2**	**41.7**	**43.3**		**28.4**	**26.4**	**21.1**	
	(37.3 - 47.1)	(36.9 - 46.7)	(32.9 - 54.2)		(24.4 - 32.7)	(22.7 - 30.3)	(15.1 - 28.2)	

**Sexual aggression**								

Victim	**2.5**	**3.6**	**3.3**		**6.6**	**8.9**	**9.9**	
	(1.2 - 4.5)	(2.1 - 5.9)	(0.7 - 9.4)		(4.6 - 9.2)	(6.6 - 11.6)	(5.8 - 15.6)	
Perpetrator	**2.2**	**2.7**	**1.1**		**0.4**	**0.2**	**ND**	
	(1.0 - 4.1)	(1.3 - 4.7)	(0.0 - 6.0)		(0.1 - 1.5)	(-0.2 - 0.5)		
Witness	**7.7**	**8.3**	**11.8**	***	**3.8**	**4.2**	**5.9**	***
	(5.5 - 10.4)	(6.1 - 11.0)	(7.3 - 17.8)		(2.6 - 5.1)	(3.0 - 5.3)	(3.6 - 9.1)	
	n = 408	n = 412	n = 90		n = 482	n = 542	n = 161	

Medellin, Colombia. 2007

*P < 0.05 ** p < 0.01 *** p < 0.001

NA: Does not apply

ND: No data

## Discussion

The most frequent studies about violence in representative community samples address the issue of victimization. The United Nations Interregional Crime and Justice Research Institute (UNICRI) has carried out several victimization surveys in representative samples of persons 16 years of age or older in urban populations in different continents. Unfortunately, it is impossible to compare the results from the present survey with those of the UNICRI surveys, since the UNICRI data refer to the last year, while items on the most severe forms of violence in the present survey refer to the respondent's lifetime [15-20]. The populations with the highest risk of victimization were men (except for robbery and sexual assault), young people, and those without a partner. Our findings with respect to being a victim of armed robbery and aggression with blunt objects and firearms are consistent with the victimization range reported by the ACTIVA survey carried out by the Pan American Health Organization in several Latin American cities and Madrid, Spain [21-23]. It is also worth noting that the armed assault figures reported in the current study are higher than the ones observed in the Epidemiologic Catchment Area Study of the U.S. National Institute of Mental Health, which states that "110 per 10,000 respondents sampled had ever used a weapon like a stick, knife or gun in a fight since the age of eighteen" [24].

There are few population studies that include witnessing violence. United States youth and their parents reported witnessing a homicide (6% and 3%), stabbing (19% and 4%) or shooting (26% and 7%) at markedly different degrees [25]. Violence witnessing ranges vary greatly among U.S. youngsters according to the group studied. For instance, Buka et al. state that youth living in American cities witnessed a great deal of violence in their communities, and that children and adolescents who report having witnessed murder ranged from a low of 1% in a "resort" group--middle- and upper-class, predominantly Caucasian youth to 47% in a low-income, predominantly African-American community. Variability was less in studies assessing predominantly low-income, urban youth, where witnessing murder was reported by one-quarter of the participants. The proportion who reported witnessing stabbings in their lifetime ranged from 9% in an affluent sample to 56% among the central-city summer camp population. The percentage of those witnessing a shooting sometime during their life ranged from 4% to 70%; among urban youth surveyed [26]. In Ontario, Canada, 34% of the population reported having been witness to aggression without a weapon during the last year, which is substantially lower than the percentage of the current study (42%) [27].

This study shows that aggression, victimization and being witness to violence are not randomly distributed in the population, but rather that certain groups are indeed more likely to have experience with one or more of these phenomena. These data are useful in defining appropriate aggression prevention policies, by calling attention to the observation that perpetrators are most likely to be young males from middle and low socioeconomic strata who have attended high school. It is also important to know the characteristics of victims, which tend to be young men with higher education --except when it comes to sexual violence--with the specific type of aggression varying in accordance with socioeconomic status. This study shows that there are more victims per perpetrator for the most severe forms of violence than for the less severe, which is in accordance with the reports that severe perpetrators make up around 5% of the population, but are responsible of 50% or more of the worst aggressions [28].

It would be advantageous for authorities to include, along with victims' characteristics, an extensive report of perpetrators' profiles in the epidemiologic surveillance system for violence. Victimization studies allow more precise estimation of the magnitude and distribution of the different forms of violence. Aggression studies allow identification of risk and protective factors associated with perpetrators, which should be the primary public health concern insofar as it is the perpetrators that produce violence and not the victims. This in turn enables the design of evidence-based policy for violence prevention and the promotion of coexistence, as has been done in Medellin and the surrounding metropolitan area. We also suggest that the UNICRI surveys include information on perpetrators and, if possible, on witnesses.

The figures we present for Medellin are indicative of a reduction in the majority of types of violence when compared with data from a similar study of 2003-2004 [4]. However, it is prudent to point out that this research, like all cross-sectional survey studies, has its limitations. Responses may have been affected by recall bias and feelings of uneasiness produced by answering questions of such seriousness in residential areas marked by high levels of aggression. It must also be noted that the sample did not include institutionalized individuals such as those found in jails, prisons, the military, and convents. Some of these groups are reportedly much more likely to report episodes of violence and victimization than are non-institutionalized residents. However, the statistical effect of including these groups in the analysis cannot be verified. Thus, caution must be exercised when drawing conclusions; the results cannot be generalized to institutionalized populations. Nonetheless, we speculate that the indices of violence reported in this study are underestimated due to the non-inclusion of these populations.

The strengths of the study are also worth highlighting. The current study casts light on an important phenomenon in a developing country. Secondly, it allows for the international comparison of data, since it employs the WHO global population standard (15-60 years of age) in the calculation of adjusted rates (Table 2). The fact that the 9% no-response rate was lower than that usually reported in this type of study and that the lack of answers was not concentrated in any particular group allows us to deduce that the conclusions are not overly biased. This study estimates the prevalence proportions of having been a witness, as well as a victim and perpetrator, which gives new perspectives on the knowledge of the distribution of violence in communities, since cross-sectional surveys carried out on random population samples tend to focus on the study of victims. The formulation of public policies for the prevention and control of violence would be better founded on scientific evidence that includes the distributions and characteristics of perpetrators and witnesses as well those of victims.

## Conclusions

Despite having registered a nearly 90% decline in homicides over the last fifteen years, Medellin continues to have one of the highest rates of homicide in Latin America. Aggression is not randomly distributed. Men reported the highest prevalence of being victims, perpetrators and witnesses in all forms of violence, except for robbery and sexual violence. The number of victims per perpetrator was positively correlated with the severity of the type of violence. The highest victimization proportions over the previous twelve months occurred among minors. Perpetrators are typically single males of lower socioeconomic strata.

Periodic surveys should be included in systems for epidemiological monitoring of violence, not only of victimization but also on perpetrators, in order to both quantify the magnitude of different forms of violence and estimate risk and protective factors that can help in the formulation of policies for violence prevention. To this end, UNICRI could include information on the characteristics of aggression and perpetrators in its surveys.

## Competing interests

The authors declare that they have no competing interests.

## Authors' contributions

LFD conceived the study, and leaded its design and coordinated its implementation and helped to draft the manuscript; AR participated in its design and coordination and was responsible to draft the manuscript, and NM participated in the design of the study and performed the statistical analysis.

All authors read and approved the final manuscript

## Pre-publication history

The pre-publication history for this paper can be accessed here:

http://www.biomedcentral.com/1471-2458/11/628/prepub

## References

[B1] Murray CJ Lopez AD The global burden of disease 1996 Cambridge, MA: Harvard University Press

[B2] Duque LF La violencia en el Valle de Aburrá. Su magnitud y programa para reducirla 2005 Medellin: Fotográficas Mario Salazar

[B3] Duque LF Klevens J Ramirez C Cross-sectional survey of perpetrators, victims, and witnesses of violence in Bogotá, Colombia J Epidemiol Community Health 2003 57 355 360 10.1136/jech.57.5.355 12700220 PMC1732461

[B4] Duque LF ed La violencia en el Valle de Aburrá. Caminos para la superación 2009 Universidad de Antioquia y Area Metropolitana del Valle de Aburrá. Medellin: Cátedra litográfica

[B5] Instituto Nacional de Medicina Legal y Ciencias Forenses Homicidios 2006 Forensis 2005 2006 Bogotá: Instituto Nacional de Medicina Legal y Ciencias Forenses

[B6] Acero AP Homicidios 2009 Forensis 2009 2010 Instituto Nacional de Medicina Legal y Ciencias Forenses 17 68

[B7] Farrington D Jolliffe D Comparing delinquency careers in Court records and self-reports Criminology 2003 41 3 933 958 10.1111/j.1745-9125.2003.tb01009.x

[B8] Kirk DK Examining the Divergence Across Self-report and Official Data Sources on Inferences About the Adolescent Life-course of Crime J Quant Criminol 2006 22 107 129 10.1007/s10940-006-9004-0

[B9] Brown DW Economic value of disability adjusted life years lost due to violence estimates for WHO Member States Rev Panam Salud Publica 2008 24 3 205 209 10.1590/s1020-49892008000900007 19115548

[B10] Duque LF Klevens J La violencia en Itagüí, Antioquia Prevalencia y distribución Biomedica 2000 20 151 168

[B11] Buss AH The psychology of aggression 1961 New York: John Wiley

[B12] Fournier M de los Rios R Orpinas P Piquet-Carneiro L Multicenter study Cultural norms and attitudes toward violence in selected cities of Latin America and Spain (ACTIVA Project): Methodology Pan American Journal of Public Health 1999 5 222 31 10355322 10.1590/s1020-49891999000400006

[B13] Straus MA Measuring Intrafamily Conflict and Violence. The Conflict Tactics (CT) Scales Journal of Marriage and Family 1970 41 75 88

[B14] Departamento Nacional de Estadística: Colombia Proyecciones de Población departamentales y municipales por área 2005 - 2020 Internet Consulted 2010, July 22th http://www.dane.gov.co/files/investigaciones/poblacion/proyepobla06_20/7Proyecciones_poblacion.pdf

[B15] OPS La Estandarización: Un Método Epidemiológico Clásico para la Comparación de Tasas Bol Epi OPS 2002 23 3 9 12

[B16] Alvazzi del Frate A Victims of Crime in the Developing countries 1998 Rome: UNICRI

[B17] Van Dijk JJM Manchin R Van Kesteren JN Hideg G The Burden of Crime in the EU, A Comparative Analysis of the European Survey of Crime and Safety (EU ICS) 2005 2007 Brussels: Gallup Europe

[B18] Van Dijk JJM Manchin R Van Kesteren JN Hideg G The Burden of Crime in the EU, A Comparative Analysis of the European Survey of Crime and Safety (EU ICS) 2005 2007 Brussels: Gallup Europe

[B19] Van Dijk JJM van Kesteren JN Smith P Criminal Victimization in International Perspective, Key findings from the 2004-2005 ICVS and EU ICS 2008 The Hague: Boom Legal Publishers

[B20] Van Kesteren J Mayhew P P N Criminal victimization in seventeen Industrialized countries. Key findings from the 2000 International Crime Victims Survey 2000 The Hague: Ministry of Justice, WODC

[B21] Cruz JM La victimización por violencia urbana: niveles y factores asociados en ciudades de América Latina y España Pan Am J Public Health 1999 5 4 259 267 10355325

[B22] Cruz JM La victimización por violencia urbana: niveles y factores asociados en ciudades seleccionadas de América Latina y España 1999 Washington: OPS 10355325

[B23] Orpinas P ¿Quién es violento? Factores asociados con comportamientos agresivos en ciudades seleccionadas de América Latina y España 1999 Washington: OPS

[B24] Swanson JW Holzer CE Ganju VK Violence and psychiatric disorder in community: evidence from the epidemiologic catchment area survey Hosp Community Psychiatric 1990 41 7 761 770 10.1176/ps.41.7.761 2142118

[B25] Kuo M Molher B Raudenbush SW Earls FJ Assessing exposure to violence using multiple informants: Application of hierarchical linear modeling J Child Psychol Psychiatry 2000 41 1049 1056 10.1111/1469-7610.00692 11099121

[B26] Buka SL Stichick TL Birdthistle I Earls FJ Youth exposure to violence: prevalence, risks, and consequences American Journal of Orthopsychiatry 2001 71 3 298 310 11495332 10.1037/0002-9432.71.3.298

[B27] Pernanen K Alcohol in human violence 1991 Nueva York: Guilford

[B28] Thornberry TP Krohn MD Taking stock of delinquency. An overview of findings from contemporary longitudinal studies 2003 Nueva York: Kluwer Academic, Plenum Publishers

